# Miniaturized Bandpass Filter Using a Meandered Stepped-Impedance Resonator with a Meandered-Line Stub-Load on a GaAs Substrate

**DOI:** 10.1155/2014/809809

**Published:** 2014-10-20

**Authors:** Z. Chuluunbaatar, C. Wang, N. Y. Kim

**Affiliations:** RFIC Center, Department of Electronic Engineering, Kwangwoon University, 447-1 Wolgye-dong, Nowon-ku, Seoul 139-701, Republic of Korea

## Abstract

This paper reports a compact bandpass filter with improved skirt selectivity using integrated passive device fabrication technology on a GaAs substrate. The structure of the filter consists of electromagnetically coupled meandered-line symmetric stepped-impedance resonators. The strength of the coupling between the resonators is enhanced by using a meandered-line stub-load inside the resonators to improve the selectivity and miniaturize the size of the filter. In addition, the center frequency of the filter can be flexibly controlled by varying degrees of the capacitive coupling between resonator and stub-load. To verify the proposed concept, a protocol bandpass filter with center frequency of 6.53 GHz was designed, fabricated, and measured, with a return loss and insertion loss of 39.1 dB and 1.63 dB.

## 1. Introduction

The importance of satellite communication systems, being the best solution to fulfill the increasing demand of global wireless communication systems, is increasing at a tremendous rate. In these systems, bandpass filters (BPFs) are used in RF front-end receivers to achieve a high image rejection ratio. The compact size and high selectivity are the most important requirements for these filters. To achieve these requirements, hairpin-line BPFs are often used because of their compact size and the ability to control the response of the filters [1–5]. Split-ring resonators with parallel-coupled lines have been introduced to miniaturize the size of hairpin-line BPFs [6]; in addition, the center frequency of such BPFs can be controlled by varying the length of the coupled portions. Additionally, via-grounded techniques have been implemented in hairpin-line structures to utilize the improved inner-stage coupling for BPFs of miniaturized size [7–9]; however, these techniques are associated with complicated patterning and drilling processes. The size of the hairpin-line BPFs employing split-ring resonators can be further reduced by increasing the length of the inner coupled lines and adding internal stub-loads. Moreover, the replacement of printed circuit board (PCB) technology used for implementing the aforementioned BPFs by integrated passive device (IPD) technology can greatly reduce the circuit size and avoid the use of via. In addition, the use of IPD process enables the integration of the BPFs with other active components of the RF front-end receivers on a single semiconductor chip and, therefore, is desirable for realizing a highly miniaturized BPF.

In this paper, we report a miniaturized hairpin BPF based on cross-coupled meandered-line stepped-impedance resonators with high selectivity by IPD technology. The size of the filter was miniaturized geometrically by folding the stepped-impedance resonator in terms of width. In addition, the dimension was further miniaturized by enhancing the coupling strength between the resonators by using an internally embedded meandered-line stub-load. The extra advantages that arise from the use of stub-load are the improved skirt selectivity and the flexibility in varying the center frequency of the filter. For application in a front-end receiver of a satellite communication system, a BPF with measured center frequency of 6.53 GHz and 3 dB fractional bandwidth of 13.7% was designed using the proposed method and fabricated on a Gallium Arsenide (GaAs) substrate. The measured return and insertion losses of the filter are 39.1 dB and 1.63 dB, respectively.

## 2. Analysis of the Proposed Resonator 

**Figure 1:**
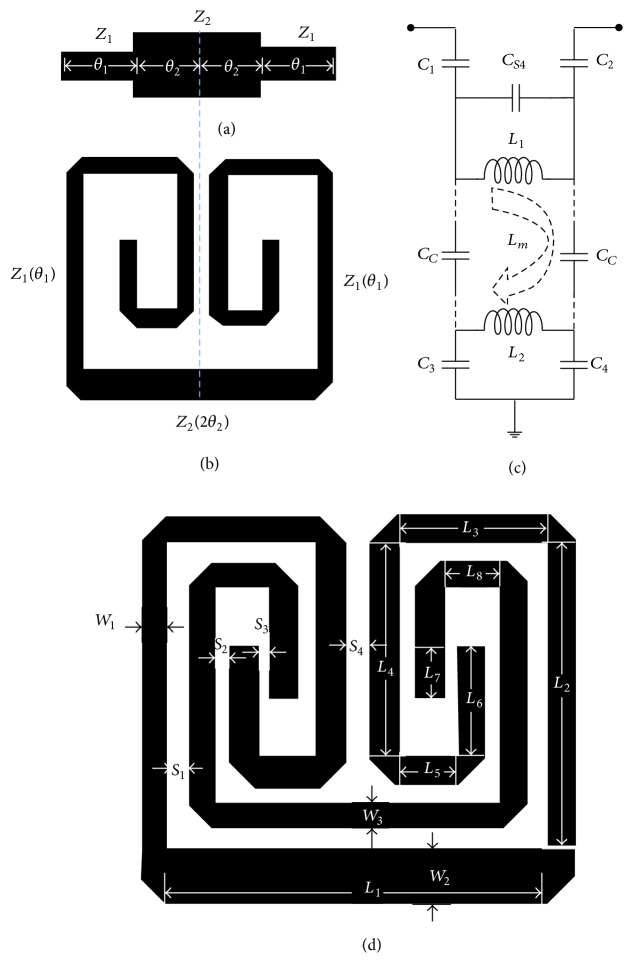
Configuration of (a) symmetric two-section SIR, (b) symmetric two-section meandered-line SIR, (c) equivalent circuit of the proposed meandered-line SIR with a meandered-line stub-load, and (d) meandered-line SIR with a meandered-line stub-load.

The configurations of the general symmetric type, two-section stepped-impedance resonator (SIR), and the meandered-line symmetric-type stepped-impedance resonator (MLSTSIR) with characteristic impedances and electrical lengths of (*Z*_1_, *θ*_1_) and (*Z*_2_, *θ*_2_) are depicted in Figures 1(a) and 1(b), respectively. The input impedance of the MLSTSIR, *Z*_*i*_ = (1/*Y*_*i*_), can be expressed as [13]

(1) $$\begin{array}{c} Z_{i}=\frac{1}{Y_{i}}=jZ_{1}\frac{Z_{2}\tan\theta_{2}+Z_{1}\tan\theta_{1}}{Z_{1}-Z_{2}\tan\theta_{2}\tan\theta_{1}}, \end{array}$$

where *Y*_*i*_ is the input admittance. The resonance condition *Y*_*i*_ = 0 gives

(2) $$\begin{array}{c} \tan\theta_{2}\tan\theta_{1}=\frac{Z_{1}}{Z_{2}}, \end{array}$$

when *θ*_1_ = *θ*_2_ = *θ*_*f*_. The resonance condition can be further simplified to obtain electrical length (*θ*_*f*_) corresponding to the fundamental resonance frequency (*f*_*f*_) as

(3) $$\begin{array}{c} \theta_{f}=\text{ta}{\text{n}}^{-1}\sqrt{\frac{Z_{1}}{Z_{2}}}. \end{array}$$

Equations (2) and (3) signify that the resonance frequency of the MLSTSIR depends on both the impedances and the electrical lengths of the resonator sections. The condition of *θ*_1_ = *θ*_2_ is generally used to simplify the analysis and obtain the minimum total electrical length of the symmetric dual-section SIR for a fixed resonant frequency [14]. However, there are always some harmonic frequencies associated with the SIR. These harmonic frequencies appear as spurious signals in the stop band of the bandpass filter to degrade the out-of-band performance. To avoid the effect of the presence of these spurious frequency signals, the distance between fundamental resonant frequency and second harmonic frequency must be maintained as large as possible, which can be achieved by using the optimized condition of *θ*_1_ ≠ *θ*_2_ for a fixed total electrical length of the SIR, which is utilized in the proposed resonator. The MLSTSIR is internally loaded with a meandered-line stub to obtain the proposed meandered-line stub-loaded SIR (MLSLSIR), which is illustrated in Figure 1(d). Figure 1(c) shows the equivalent circuit of the MLSLSIR. *C*_*S*4_ represents the capacitive coupling between the folded arms of the MLSTSIR, which is represented by *C*_1_-*L*_1_-*C*_2_ in the circuit. *C*_*c*_ and *L*_*m*_ account for capacitive and inductive coupling between the MLSTSIRs and stub-load which is represented by *C*_3_-*L*_2_-*C*_4_.

## 3. Bandpass Filter Design 

**Figure 2:**
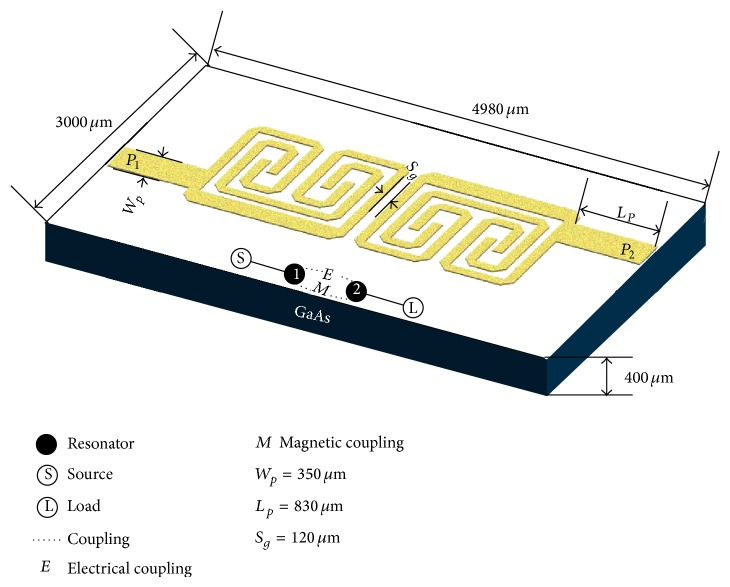
The layout of the proposed BPF including the coupling scheme between the resonators.

**Figure 3:**
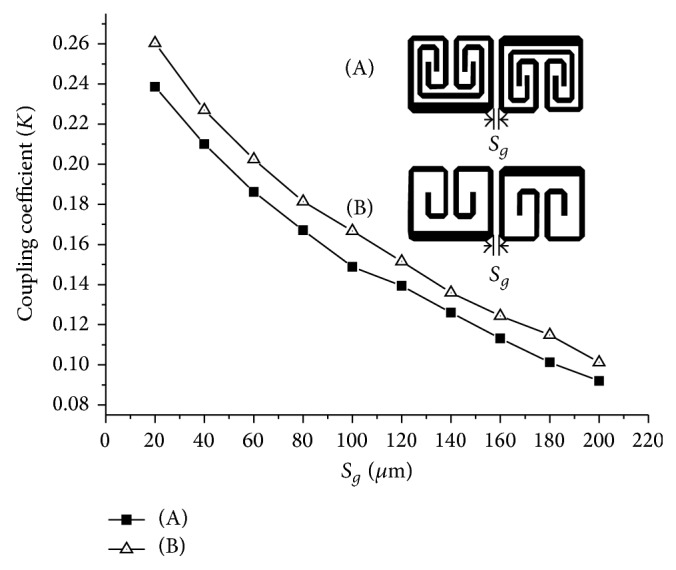
The coupling coefficient as a function of the spacing *S*_*g*_ between the resonators, (A) the stub-load meander-line and (B) without the stub-loaded meander-line.

**Figure 4:**
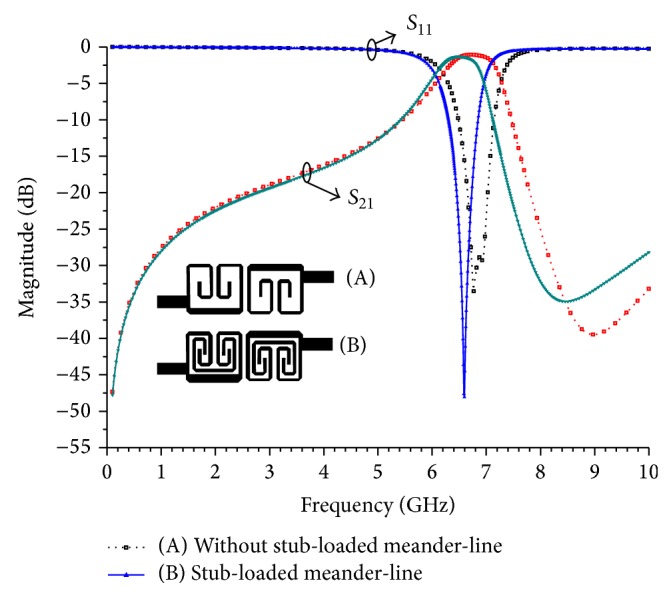
The comparison of the simulated structure, (A) without the stub-loaded meander-line and (B) the stub-load meander-line.

**Figure 5:**
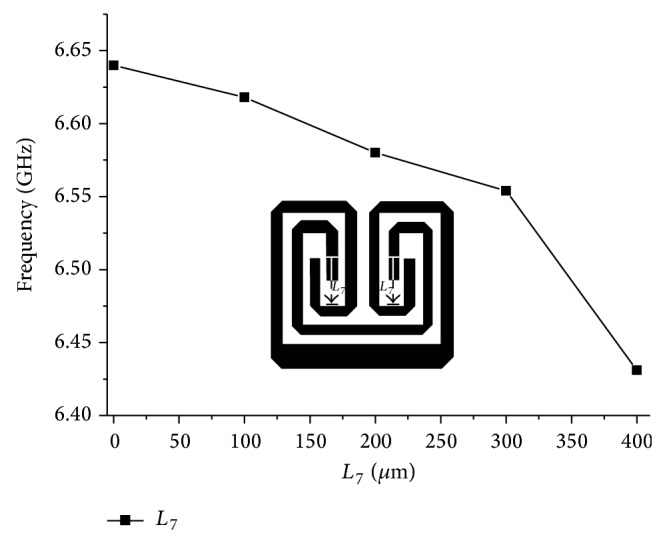
The frequency shifting to the lower-side by varying the *L*_7_ overlapping open-ends of the meandered stubs.

To obtain a BPF with a center frequency of 6.6 GHz based on the proposed resonator, two MLSLSIRs connected with 50 Ω transmission lines are coupled with each other using mixed coupling. The MLSTSIR with optimized parameters of *Z*_1_ = 72.3 Ω, *θ*_1_ = 72.8°, *Z*_2_ = 57.5 Ω, and *θ*_2_ = 31.4° is loaded with 72.3 Ω meandered-line stub of electrical length of 85.5°.  Figure 2 shows the layout of the proposed BPF and illustrates the coupling topology of the resonators.  Figure 3 depicts the electromagnetic coupling structure and coupling coefficients as function of coupling gap between the meandered-line stub-load and the unloaded resonators. The coupling coefficient can be determined as follows [15]:

(4) $$\begin{array}{c} K=\frac{f_{2}^{2}-f_{1}^{2}}{f_{2}^{2}+f_{1}^{2}}, \end{array}$$

where *f*_1_ and *f*_2_ are the first and second resonant frequencies, respectively. The coupling gap *S*_*g*_ is varied from 20 *μ*m to 200 *μ*m. Two separated resonant frequencies are obtained and the coupling coefficient represents the electromagnetic coupling effect of the two resonators. The inserted meandered-line stub-load also reduces the size of the layout in two ways compared to the unloaded structure. First, the layout size is reduced via the capacitance effect between the meandered-line stub-load and open-end of the resonator, which decreases the resonant frequency. Second, the layout size is reduced because the meandered-stub weakens the coupling between these resonators considerably as shown in Figure 3. The resonators will be much closer for the same coupling coefficient. By optimizing the *S*_*g*_ parameter, both the bandwidth and the return loss can be precisely controlled. The proposed design used *S*_*g*_ that is equivalent to 120 *μ*m at 6.6 GHz. A comparison of the meandered-stub loaded and unloaded structure is depicted in Figure 4. When the meandered-line stub-load was inserted, the center frequency downshifted from 6.87 GHz to 6.6 GHz and the return loss improved from 28.9 dB to 48.1 dB due to the increasing capacitive coupling effect between the two open-ends.  Figure 5 shows that the effect of the overlapping distance *L*_7_ can be used to achieve the desired frequency response. As the overlapping distance varies from 0 *μ*m to 400 *μ*m, the operating frequency is obviously shifted to lower frequencies by increasing the degree of the capacitive coupling while maintaining the overall dimension of the BPF.

## 4. Implementation and Measurement

**Figure 6:**
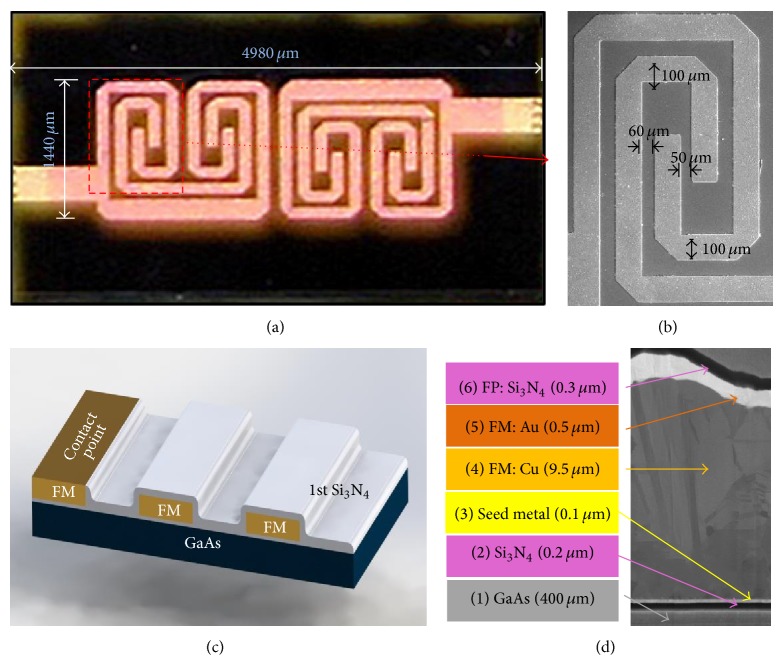
The enlarged image of the proposed BPF: (a) enlarged view of the layout. (b) SEM view of the emphasised region. (c) 3D cross-sectional view of the IPD process on GaAs. (d) FIB cross-sectional image of the BPF.

**Figure 7:**
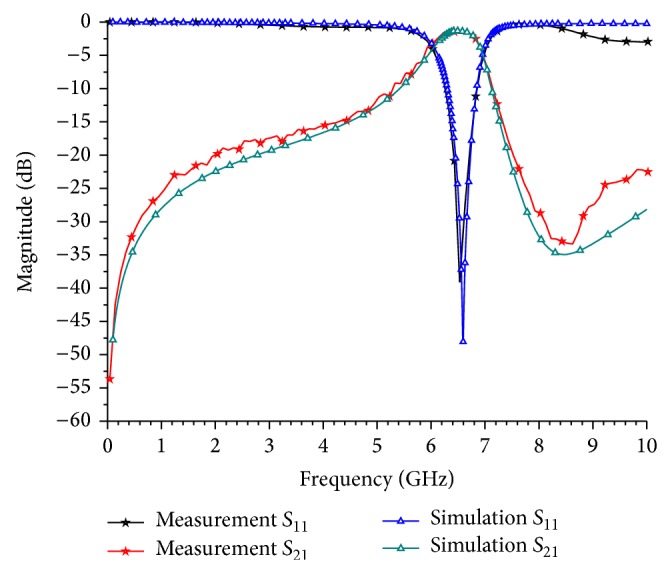
Simulated and measured results of the proposed BPF.

To verify the application of the proposed MLSLSIR in the BPF design, a filter with a centre frequency of 6.6 GHz was designed and fabricated on a conventional 6-inch, 400 *μ*m thick GaAs substrate (with dielectric constant *ε*_*r*_ = 12.85 and loss tangent = 0.006) following the standard IPD fabrication process [16, 17]. The simulation and optimisation of the filter were performed using a full wave EM Sonnet simulator, and the optimised physical dimensions of the MLSLSIR shown in Figure 1(d) are *L*_1_ = 1400 *μ*m, *L*_2_ = 1140 *μ*m, *L*_3_ = 550 *μ*m, *L*_4_ = 800 *μ*m, *L*_5_ = 220 *μ*m, *L*_6_ = 410 *μ*m, *L*_7_ = 190 *μ*m, *L*_8_ = 210 *μ*m, *W*_1_ = 100 *μ*m, *W*_2_ = 200 *μ*m, *W*_3_ = 100 *μ*m, *S*_1_ = 70 *μ*m, *S*_2_ = 60 *μ*m, *S*_3_ = 50 *μ*m, and *S*_4_ = 100 *μ*m. The BPF was designed using two coupled MLSLSIRs, as illustrated in Figure 2, with the optimised coupling gap of *S*_*g*_ = 120 *μ*m. The fabricated BPF was mounted onto an FR-4 printed circuit board (PCB) with two ports calibrated using 50 ohm impedance matching for the RF measurements and the actual device is shown in Figure 6(a). An image of the emphasised region taken by scanning electron microscope (SEM) is shown in Figure 6(b). A 3D cross-sectional view of the IPD process on a GaAs substrate and the focused ion beam (FIB) cross-sectional image are shown in Figures 6(c) and 6(d), respectively. The center frequency of the fabricated filter tested and characterized using an Agilent 8510C vector network analyser (VNA) is 6.53 GHz, which is downshifted by 70 MHz with respect to the simulation result. This frequency shift can be attributed to the dielectric loss of the substrate, the dispersion loss at the bends of MLSLSIRs, and the accuracy of the physical dimensions. The measured insertion and the return losses at the centre frequency were 1.63 dB and 39.1 dB, respectively. Comparison between measured and simulated results is in good agreement as shown in Figure 7. The observed minimum return loss of 14.5 dB in the stop band of the BPF from 0.1 GHz to 4 GHz and from 7.4 GHz to 12.3 GHz indicates that the filter has a wide stop band performance. The 3 dB fractional bandwidth of the pass band was measured to be 13.7%. As presented in Table 1, this filter has a very compact size in terms of *λ*_*g*_ and the highest return loss compared to those reported in other works.

## 5. Conclusion

In this paper, a miniaturized narrow-band BPF based on MLSLSIRs using IPD technology was designed, simulated, and fabricated on GaAs substrate. The physical size of the filter was miniaturized by using embedded stub-loads, which improved the strong coupling effect between the coupled resonators. The designed BPF operates at the measured centre frequency of 6.53 GHz with a corresponding fractional bandwidth of 13.7% and has a compact size of 0.31*λ*_*g*_ by 0.08*λ*_*g*_ with an excellent return loss of 39.1 dB. Compared with the results of other research works, the proposed MLSLSIRs BPF has the advantages of compact and miniaturized size with a sharp and deep skirt, which is compatible for satellite RF front-end receiver applications.

## Figures and Tables

**Table 1:** Comparison with other reported BPFs.

	Center frequency (GHz)	Insertion loss (dB)/return loss (dB)	*ε*_*r*_, *h* (mm)	Size (*λ*_*g*_ × *λ*_*g*_)
[10]	2.28	1.2/10	9.5, 0.635	0.21 × 0.15
[11]	1.5	2.9/16	3.38, 0.508	0.97 × 0.23
[12]	1.45	1.9/18	3.38, 0.508	0.18 × 0.17
This work	6.53	1.63/39.1	12.85, 0.4	0.31 × 0.08
